# Risk factors for catheter-related infections in patients receiving permanent dialysis catheter

**DOI:** 10.1186/s12882-019-1392-0

**Published:** 2019-05-31

**Authors:** Fani Delistefani, Manuel Wallbach, Gerhard A. Müller, Michael J. Koziolek, Clemens Grupp

**Affiliations:** 1 Department of Nephrology and Rheumatology, University Medical Center Göttingen, Georg-August-University, Robert-Koch-Str.40, 37099 Göttingen, Germany; 2 Department of Nephrology and Hypertension, Academic Teaching Hospital Bamberg, Bugerstrasse 80, 96049 Bamberg, Germany

**Keywords:** Catheter related complication, CRP, Germ carriage, Hemodialysis, MRSA, Tunneled catheter

## Abstract

**Background:**

Due to rising vascular comorbidities of patients undergoing dialysis, the prevalence of permanent hemodialysis catheters as hemodialysis access is increasing. However, infection is a major complication of these catheters. Therefore, identification of potential predicting risk factors leading to early infection related complications is valuable, in particular the significance the CRP (C-reactive protein)-value is of interest.

**Methods:**

In this retrospective study 151 permanent hemodialysis catheters implanted in 130 patients were examined. The following data were collected at the time of catheter implantation: CR*P*-value, history of catheter-related infection, microbiological status, immunosuppression and diabetes mellitus. The primary outcomes were recorded over the 3 months following the implantation: catheter-related infection, days of hospital stay and death. Catheter removal or revision, rehospitalization and use of antibiotics were identified as secondary outcomes.

**Results:**

We identified a total of 27 (17.9%) infections (systemic infection: 2.26 episodes/ 1000 catheter days, local infection: 0.6 episodes/ 1000 catheter days). The development of an infection was independent of the CRP-value (*p* = 0.66) as well as the presence of diabetes mellitus (*p* = 0.64) or immunosuppression (*p* = 0.71). Univariate analysis revealed that infection was more frequent in patients with MRSA-carriage (*p* < 0.001), in case of previous catheter-related infection (*p* < 0.05) and of bacteremia or bacteriuria in the period of 3 months before catheter implantation (*p* < 0.001). Catheter removal or revision (*p* = 0.002), rehospitalization (*p* = 0.001) and use of antibiotics (*p* = 0.02) were also more often observed in patients with MRSA-carriage.

**Conclusions:**

The CRP-value at the time of implantation of a permanent hemodialysis catheter is not associated with the development of early catheter related infections, but an individual history of catheter-related infection, MRSA-carriage and bacteremia or bacteriuria in the period of 3 months prior to catheter implantation are significant risk factors.

## Background

Although best vascular access for hemodialysis treatment is an arteriovenous fistula, the prevalence of permanent hemodialysis catheters as hemodialysis access among patients with end-stage renal disease is increasing [1] because of the need for temporarily access during acute kidney injury or long maturation of arteriovenous accessor when opportunities for an arteriovenous access are exhausted. The advantages of permanent central venous catheters are their ability to be inserted quickly and easily and allow immediate access for hemodialysis, the atraumatic and thus painless connection to dialysis and the missing cardiac volume load.

The disadvantage, however, is a significantly higher complication rate than with other options of vascular access, such as arteriovenous fistulae and synthetic vascular grafts [2]. Permanent hemodialysis catheters are associated with lower dialytic efficacy compared to arteriovenous fistulae [3]. Moreover, there is an increased incidence of mechanical dysfunction [4], thrombosis [5] and bacteremia [6]. Related complications, especially infections, have unfavorable effects on morbidity, mortality and costs. Infections are the second most frequent cause of death in dialysis patients and therefore play a major role since they lead to about 1300 hospitalizations per 1000 patient years [7]. For tunneled-cuffed catheters infection rates of about 0.5–5.5 events per 1000 catheter-days have been reported [4]. Data from the US renal data registry showed that between 1993 and 2014 the rate of hospitalization due to infections in hemodialysis patients rose by 34% [8]. The infectious complications occur especially in the first 3 months after catheter implantation [9]. Conceivable is, that factors already present at the time of implantation might be associated with an increased risk of early catheter related infections. As one approach to reduce this risk, identification of factors leading to infectious complications is required.

In this study we investigated the significance of the C-reactive protein (CRP), but also the germ carriage status and/or a history of a recent bacterial infection at the time of implantation of a permanent dialysis catheter as risk factors for the development of catheter-related infections and potentially associated outcome parameters in these patients.

## Methods

### Study design, inclusion/exclusion criteria and ethics

Our study is a retrospective evaluation of medical records of patients in whom a permanent tunneled atrial catheter for hemodialysis was implanted in the period from 01.01.2004 to 31.12.2013 in the University Medical Centre Göttingen, Germany. A tunneled atrial catheter system was necessary as bridging to shunt maturation or in patients who were unable to get an arterio-venous (AV)-fistula system for various reasons. The tunneled catheters were inserted by surgeons of the Department of thoracic, heart and vascular surgery either into the internal jugular or subclavian vein.

Exclusion criteria for our study were the rejection of the data analysis or a lack of consent from the patient as well as an active tumor disease at the time of the catheter implantation.

The study was approved by the local Ethical Committee of Göttingen. The investigation conforms to the principles outlined in the Declaration of Helsinki.

### Data analysis and endpoints

The following data were collected at the time of catheter implantation: age, gender, CRP value (on the day of implantation or, if not available, in the period of 2 days before until 2 days after the day of catheter implantation (no significant differences between these were observed), previous catheter related infection, microbiological results (like germ carriage of MRSA), infection with germ detection in a blood or urine culture in a period up to 3 months before catheter implantation, localization of the catheter, previous temporary catheter, immunosuppression, diabetes mellitus and the cause of end-stage renal disease.

Primary outcomes were recorded over the 3 months following the implantation: catheter-related local infection or systemic infection, days of hospital stay and death. Catheter removal or revision, rehospitalization and use of antibiotics were considered as secondary outcomes. An isolated exit site infection was defined as erythema at the insertion site for which antibiotic therapy was instituted or a culture-positive exudate from the exit site in the setting of negative blood cultures. Systemic catheter related infections were assumed when blood cultures taken from the catheter turned positive during episodes of exit site infection or when other primary causes of infection (by physical examination, urinalysis chest radiography etc.) were negative and infection was classified as catheter related infection in the medical records.

In cases where no antibiotic therapy was administered for other reasons, periinterventional single-shot antibiosis was administered. That was not counted as reaching the endpoint.

### Statistics

Statistical analyzes were performed using STATISTICA 13.3 for Windows from StatSoft (StatSoft (Europe) GmbH, 20,253 Hamburg, Germany). Continuous variables were expressed as means and standard deviation (+ SD) or median with the 25th and 75th percentiles [interquartile range (IQR)], as appropriate. Normal distribution of the data was examined by the Shapiro-Wilk-test. Two medians were compared applying the Mann-Whitney U test, more than two by the Kruskal-Wallis ANOVA (ANalysis Of Variance). Categorial variables were evaluated using Chi^2^ or Fishers exact test. A multivariate analysis was performed using linear regression when outcome variables were continuous, and logistic regression was used when outcome variables were dichotomous, covariates were removed using backward selection. A *P*-value < 0.05 was considered as statistically significant.

## Results

### Patients

A total of 150 patients underwent the implantation of permanent central venous hemodialysis catheters over a period of 10 years. Thirteen of 130 patients were excluded due to active cancer disease, 7 patients because of a missing CRP value at the time of catheter implantation. Overall we investigated 151 permanent central venous hemodialysis catheters implanted in 130 patients, 97 into the subclavian and 54 into the intern jugular vein. In 25 cases of catheter implant there was incomplete information relating to the endpoints. Prior dialysis dependency preexisted in 24 patients (median time on dialysis: 33 [IQR 14–63] months).

The mean age of the 130 included patients was 68.8 ± 12.8 (range 25–87 years). Eighty-three of 151 catheters were implanted in male patients. In 68.9% of the cases the cause of hemodialysis was an acute or an acute on chronic kidney injury. 47.7% of the patients suffered from diabetes mellitus. The median/mean CRP value at the time of catheter implantation was 24.7[IQR 9.4–38.3]/29.3 + 26.8 mg/l (normal value < 5.0 mg/l). Patient characteristics are summarized in Table 1.

**Table 1:** Patients´ characteristics

Characteristic	CRI	No-CRI	*p* value
Total number of patients (female; male: *n* = 55; 75)	10;11	45;64	0.59
Total number of implanted dialysis catheters (in female; male: *n* = 68; 83)	13;14	55;69	0.72
Age (years)	69.1 ± 8.4	68.7 ± 13.6	0.90
Acute or acute on chronic renal injury (*n* = 93 [71.5%])	16	77	0.78
Prior dialysis dependency (*n* = 24 [18.5%])	4	20	1.00
Underlying diseases			
Diabetic nephropathy (*n* = 46 [35.4%])	9	37	0.45
Hypertensive nephrosclerosis (*n* = 33 [25.4%]	5	28	1.00
Glomerulonephritis (*n* = 16 [12.3%])	3	13	0.72
Post-operative acute renal injury (*n* = 12 [9.2%])	0	12	0.21
Cardio-renal syndrome (*n* = 7 [5.4%])	3	4	0.08
Unknown etiology (*n* = 17 [13.1%])	4	13	0.76
Others ^*^ (*n* = 25 [19.2%])	3	22	0.76
Relevant concomitant diseases			
Diabetes mellitus (*n* = 62 [47.7%])	11	51	0.64
Congestive heart failure (*n* = 82 [63.1%])	18	64	0.03
Coronary artery disease (*n* = 68 [52.3%])	12	56	0.64
Arterial hypertension (*n* = 118 [90.8%])	21	97	0.21
COPD (*n* = 19 [14.6%])	4	15	0.51
Relevant treatment			
Immunosuppression (*n* = 15 [11.5%])	3	12	0.71

Data are shown as mean (± SD) or as absolutes (percentages). ^*^Contrast-induced nephropathy (*n* = 5), nephrectomy because of renal cell carcinoma (*n* = 5), polycystic kidney disease (*n* = 4), pyelonephritis (*n* = 3), rhabdomyolysis (*n* = 2), hemolytic-uremic syndrome (*n* = 2), hydronephrosis(*n* = 2), analgesic nephropathy(*n* = 1)

*CRI* catheter related infection

### CRP value and primary and secondary outcomes

**Fig. 1:**
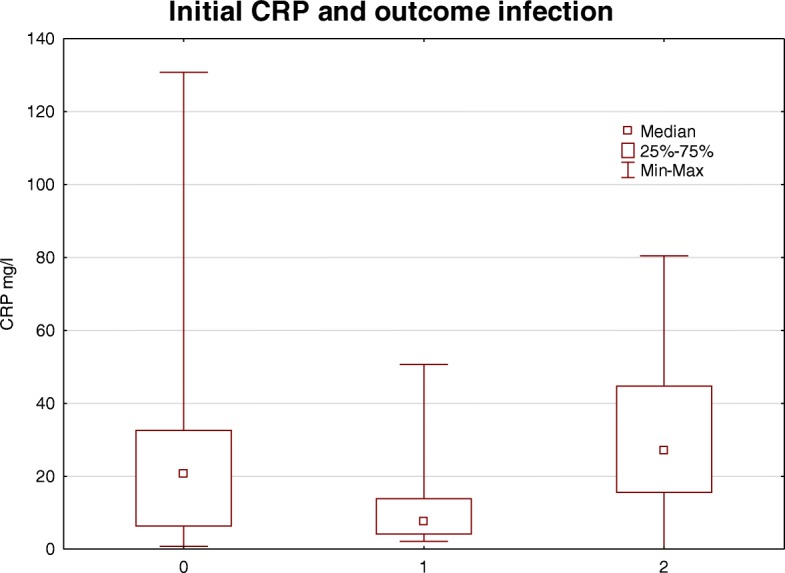
There was no significant difference between the medians of initial CRP values of patients without (0), local (1) and systemic (2) infection within 3 months after catheter implantation (*P* = 0.15)

An infection (local or systemic) was detected in 17.9% of the cases in the time of 3 months after the implantation of the catheter (systemic infection: 2.26 events/ 1000 catheter days, local infection: 0.6 events/ 1000 catheter days). The initial median CRP value of the patients without infection (20.6 [IQR 6,2–32.7] mg/dl) was not significantly different to the median CRP value of the group with local infection (7.7 [IQR 4–14] mg/dl; *P* = 0.23) and also compared to the group with systemic infection (26.9 [IQR 15–44.9] mg/l; *P* = 0.31) (Fig. 1). Univariate linear regression showed no significant association between initial CRP value and catheter related infections (*P* = 0.66).

Twenty-two patients died in the period of 3 months after the implantation of the catheter. The median initial CRP value of the group with death 29.5 [IQR15.5–43.2] was not significantly different to the median initial CRP value of the group without death 19.4 [IQR 7.1–32.7] mg/dl (*P* = 0.08). Concerning the secondary outcomes catheter removal, rehospitalization and need for use of antibiotics there was no significant difference by univariate analysis in initial CRP values either. Data are summarized in Table 2.

**Table 2:** Initial CRP value and various outcomes

	Initial CRP value [mg/l]		
Outcome	CRI	no CRI	*p*
Death (*n* = 22/104)	29.5 (15.4–43.2)	19.4 (7.1–32.7)	0.08
Catheter removal or revision (*n* = 30/96)	23.4 (9.0–41.1)	20.6 (8.1–32.7)	0.26
Rehospitalization (*n* = 34/92)	19.8 (10.6–41.1)	24.3 (8.9–32.7)	0.57
Use of antibiotics (*n* = 25/101)	23.4 (14.0–46.1)	20.6 mg/l (6.2–32.7)	0.14

Initial CRP values are shown for various primary (death) and secondary outcome parameters (frequency n with/without CRI are given in brackets). CRP values are reported as median and 25%- and 75%-quantiles, respectively

*CRI* catheter related infection

Analyzing the association between CRP value and primary and secondary outcomes by multivariate linear regression showed a significant effect exclusively with the length of the stay in hospital (*P* < 0.01). The mean duration of stay in hospital of the patients after the implantation of the catheter was 11.4 days (± 15.4). There was only a weak positive correlation in Spearman’s rank test between the initial CRP value and the duration of the stay in hospital (r_Sp_ = 0.23, *p* = 0.004).

### Additional risk factors relating to outcomes

No significant influence of the parameters age, gender, diabetes mellitus, immunosuppression, temporary dialysis catheter and localization of the permanent dialysis catheter on primary and secondary outcomes was observed by logistic regression (data not shown).

### History of catheter related infection

Fifteen patients had a history of a catheter related infection. Seven of the 15 patients (46.7%) had a catheter related re-infection within the observation period of 3 month. In the group without a history of a catheter related infection (111 cases) only 20 patients (18%) had a catheter related infection within the observation period of 3 months (*p* = 0.02) (Table 3). The probability for the appearance of a catheter related infection (local and systemic) based on the risk factor “history of catheter related infection” proved to be significant also by logistic regression (*P* = 0.03) as well as for a local catheter infection (*P* = 0.02) but not for a systemic catheter infection (n.s., not shown) (Table 4).

**Table 3:** Infection related risk factors and various outcome parameters

	Risk factor								
	MRSA-carriage			History of catheter related infection			Bacteremia or bacteriuria within 3 month before implantation		
Outcome	**yes** ***n*** **= 17**	**no** ***n*** **= 109**	***p***	**yes** ***n*** **= 15**	**no** ***n*** **= 111**	***p***	**yes** ***n*** **= 29**	**no** ***n*** **= 97**	***p***
Catheter related infection (all)	**9 (52.9%)**	**18 (16.5%)**	**< 0.001**	**7 (46.7%)**	**20 (18.0)**	**0.02**	**13 (44.8%)**	**14 (14.4%)**	**< 0.001**
Death	3 (17.6%)	19 (17.4%)	0.98	0 (0.0%)	22 (19.8)	0.13	7 (24.1%)	15 (15.5%)	0.28
Catheter removal or revision	**9 (52.9%)**	**21 (19.3%)**	**0.002**	5 (33.3%)	25 (22.5%)	0.33	10 (34.5%)	20 20.6%	0.12
Re-hospitalization	**10 (58.8%)**	**24 (22.0%)**	**0.001**	7 (47.6%)	27 (24.3%)	0.12	**14 (48.3%)**	**20 (20.6%)**	**0.003**
Use of antibiotics	**7 (41.2%)**	**18 (16.5%)**	**0.02**	6 (40.0%)	19 (17.1%)	0.08	**12 (41.4%)**	**13 (13.4%)**	**0.001**

Various outcomes depending on the risk factors MRSA-carriage, history of catheter related infection and bacteremia or bacteriuria in the period of 3 months before catheter implantation. Absolute numbers (percentage) of patients positive/negative for the respective risk factor are given. *P*-values < 0.05 are highlighted by bold letters

**Table 4:** Association between various patient factors and primary or secondary outcomes

Patient factor	Outcome	Odds ratio	95% Confidence intervals (lower-upper)	*P*-value
History of catheter related infection	Catheter related infection (local and systemic)	3.86	1.16–12.84	< 0.03
	Catheter related infection (local)	10.13	1.72–59.69	< 0.02
MRSA carriage	Catheter related infection (local and systemic)	5.94	1.99–17.74	< 0.002
	Catheter related infection (local)	7.39	1.36–40.28	< 0.03
	Catheter removal or revison	4.58	1.58–13.32	< 0.01
	Rehospitalization	4.98	1.71–14.51	< 0.005
	Use of antibiotics	3.59	1.20–10,76	< 0.05
Bacteremia or bacteriuria within 3 month before implantation	Catheter related infection (local and systemic)	5.11	1.98–13.16	< 0.001
	Rehospitalization	3.54	1.46–8.56	< 0.005
	Use of antibiotics	4.70	1.80–12.23	< 0.002

Only significant associations are shown, for details see text. Patient factors analyzed: gender, age, CRP value, immunosuppression, diabetes mellitus, previous catheter infection, germ carriage, MRSA, positive blood or urine culture up to 3 months before implantation, catheter localization

In cases with a history of catheter related infection no significant effects on the endpoints “use of antibiotics”, “death” and “catheter removal or revision” were observed by univariate analysis (Table 3).

### Methicillin resistant *Staphylococcus aureus* (MRSA)

In 17 cases (14.0%) a methicillin resistant *Staphylococcus aureus* (MRSA) before catheter implantation was detected. Within the observation period, 17.6% cases with MRSA carriage showed local catheter infection but only in 2.8% without (*p* = 0.007). The probability of the appearance of a local catheter infection based on the determining factor “MRSA carriage” was significantly increased (*p* = 0.026) also after logistic regression.

MRSA carriage was also significantly associated in univariate analyses with the endpoints systemic catheter infection, permanent dialysis catheter removal or revision, rehospitalization, and need for antibiotics use, which remained significant in all cases after logistic regression but not on the endpoint death. Results are summarized in Tables 3 and 4.

### Bacteremia or bacteriuria in the period of 3 months before catheter implantation

In 29 of 126 cases (23.0%) a germ in blood, urine or the tip of a vein catheter were detected in the time of 3 months before the implantation of the permanent dialysis catheter. In 44.8% of these cases a local or systemic catheter infection appeared, but only in 14.4% without previous germ detection (*p* < 0.001). This remained significant (p < 0.001) even after logistic regression. Moreover, in patients with germ detection the rate of rehospitalization as well as the need for antibiotics use was significantly altered, but there was no significant difference for the endpoints local catheter related infection, catheter removal or revision, death or days of hospital stay. Results are summarized in Tables 3 and 4.

## Discussion

A great international study with over 7000 hemodialysis patients revealed that catheter-related complications appear mainly in the first 3–6 months after implantation of the catheter [9, 10]. In particular infections and their associated consequences are considered as a major complication of these catheters. The infection rate of tunneled atrial catheters in our study was, with a total of 2.86 events per 1000 catheter days, comparable to the results of similar studies [11, 12]. Only few studies differentiated, as we did, between local and systemic catheter related infections [4]. The difference with respect to local catheter related infections between the latter and our study might be explained by different definitions of exit site infections.

As it is unknown which factors contribute to this clinically very important fact, our study was conducted with the aim of investigating such potential influencing factors and their potential predictive power for relevant endpoints. We limited our observation to a period of 3 months, since we assumed that the impact of these risk factors present at the time of implantation will be greatest within this early period and decrease with time due to the emerging role of other risk factors. Of particular interest in this context was the significance of the initial CRP-value at the time of implantation for these endpoints within 3 months. However, the CRP values of patients with end stage renal disease, notably of patients with diabetes mellitus [13], are in absence of an infection higher than CRP values of people without end stage renal disease [14]. This was given in our study and was comparable to other studies [13, 15, 16].

Our study showed no significant influence of the CRP value at the time of implantation of the permanent dialysis catheter on any investigated outcome. An older study identified an elevated CRP value as a risk factor for bacteremia in chronic hemodialysis patients [17] but this study was independent of catheter implantation. Another newer study examined similar to our study the influence of the CRP value at the time of implantation of the catheter on catheter-related complications in hemodialysis patients. There was also no significant influence of the CRP value on the appearance of a catheter-related bacteremia [2]. One study identified the CRP value as an independent predictor for death in chronic hemodialysis patients in a 6 year follow-up [18] and another for hospitalization in ambulant chronic dialysis patients [19]. All the cited studies, however, differed from ours in the observation period. While these were long-term examinations independent of the implantation procedure, our focus is on the short but relevant period of months after catheter implantation, to determine already existing potential risk factors at the time of implantation.

Our study identified MRSA carriage, bacteremia or bacteriuria in the period of three 3 months before catheter implantation and a history of catheter infection as relevant risk factors for development of several investigated endpoints. This is new, relevant and has not been described before. Other investigated parameters (age, gender, diabetes mellitus, immunosuppression, previous temporary dialysis catheter or localization of the permanent dialysis catheter) had no influence on endpoints. However, in a long-term observation period a significant influence of diabetes mellitus on rehospitalization and bacteremia [20, 21] and of immunosuppression on bacteremia [17] has been shown.

The rate of MRSA carriage in our study was with 13.9% comparable to other populations [22–25] and, therefore, representative. Some other studies revealed a significant influence of MRSA carriage in hemodialysis patients on MRSA-related infections [26, 27] and death [28]. Relating to the endpoint death the period of 3 months after implantation in our study seems too short to show a significant influence of MRSA carriage. A prophylactic procedure could be the MRSA eradication with mupirocin nasal ointment. In the field of surgery two greater studies exist, which investigated this topic but with contradictory results [29, 30]. Moreover, the type of procedure, cardiothoracic and orthopedic surgery, was completely different from ours. Therefore the role of mupirocin treatment remains actually unclear in this context.

Furthermore, systemic germ detection, i.e. a detection of a germ in blood or urine, 3 months before the implantation of the permanent hemodialysis catheter was identified as a risk factor for catheter-related infection, rehospitalization and the need of antibiotics treatment, but had no influence on the endpoints death, catheter removal or revision and days of hospital stay. This is in line with some previous studies identifying a history of bacteremia in hemodialysis patients as a risk factor for the development of a catheter related bacteremia [20, 31]. Data relating to a history of bacteriuria as risk factor have so far not been available to the best of our knowledge.

The limitations of our study are its monocentric and retrospective character and a relative small number of patients. However, the results, which could be compared to those of other studies, are well in agreement with the majority of previous results. This suggests that we examined a rather representative study population permitting convincing conclusions.

## Conclusions

Our study demonstrated that the CRP value at the time of implantation of permanent hemodialysis catheter was not associated with the development of catheter-related complications in the period of 3 months after implantation. In contrast, as significant risk factors we identified MRSA carriage, a history of previous catheter related infection and a history of bacteremia or bacteriuria in the period of 3 months before the implantation of the catheter. Thus, physicians should focus on the amelioration of infection prevention. Whether MRSA eradication and prophylactic or longer treatment with antibiotics in case of systemic germ detection in the last 3 months before the implantation of the catheter can reduce the incidence of adverse outcome remains to be shown.

## Data Availability

The datasets used and/or analysed during the current study are available from the corresponding author on reasonable request at the following address: mkoziolek@med.uni-goettingen.de.

## Abbreviations

ANOVA: ANalysis Of Variance
CRP: C-reactive protein
IQR: Interquartile Range
MRSA: Methicillin*-*resistant *Staphylococcus aureus*
SD: Standard deviation
